# The quality of life in Chinese population with chronic non-communicable diseases according to EQ-5D-3L: a systematic review

**DOI:** 10.1007/s11136-018-1928-y

**Published:** 2018-07-06

**Authors:** Ting Zhou, Haijing Guan, Jiaqi Yao, Xiaomo Xiong, Aixia Ma

**Affiliations:** 1 School of International Pharmaceutical Business, China Pharmaceutical University, No. 639 Longmian Avenue, Jiangning District, Nanjing, 211198 Jiangsu China; 2 China Center for Health Economic Research, Peking University, Beijing, China; 3 School of Pharmaceutical Sciences, Peking University, Beijing, China

**Keywords:** Quality of life, Chronic non-communicable diseases, EQ-5D-3L, Chinese population

## Abstract

**Purpose:**

Over the past decade, a changing spectrum of disease has turned chronic non-communicable diseases (CNCDs) into the leading cause of death worldwide. During the 2015 in China, there were more than 6.6 million deaths from NCDs, which was the highest rate around the world. In the present study, we performed a systematic review to analyze the health-related quality of life (HRQoL) according to EQ-5D-3L instrument in patients with different kinds of CNCDs in China.

**Methods:**

We searched PubMed, Embase, Web of Science, Cochrane Library, VIP, WanFang Data, and CNKI databases up to April 12, 2018, to identify all relevant studies that reported on HRQoL assessed by EQ-5D-3L instrument in Chinese patients with CNCDs. Expert consultation and hand-searching of reference lists from retrieved studies were employed to identify additional references. The variation of mean utility values, EQ-VAS score ranges, and responses for each EQ-5D dimension described in relevant studies were extracted.

**Results:**

A total of 5027 English-language articles and 618 Chinese-language articles were identified, among which 38 articles met full inclusion criteria. These 38 studies involved 18 kinds of CNCDs. In this review, the health utility for diabetes mellitus ranged from 0.79 to 0.94 (EQ-5D VAS scores from 61.5 to 78.6), hypertension from 0.78 to 0.93 (70.1–77.4), coronary heart disease from 0.75 to 0.90 (71.0–77.0), chronic obstructive pulmonary disease from 0.64 to 0.80 (55.0–67.0), epilepsy from 0.83 to 0.87 (78.3–79.6), cerebral infarction from 0.51 to 0.75 (49.7–79.0), while children cerebral palsy was 0.44 (27.3).

**Conclusions:**

EQ-5D-3L is widely used in studies of HRQoL associated with CNCDs in China. Our results suggest that many factors may influence the measurement results of health utilities, including age, gender, sample source, comorbidities, rural/urban, and EQ-5D-3L value sets.

## Introduction

There are more than 1.3 billion people in China, which make almost 1/6 of world’s population, and largely contribute to a global patients’ community. During the 2015 in China, there were more than 6.6 million deaths from non-communicable diseases (NCDs), which was the highest rate around the world [1]. Over the past decade, diseases such as diabetes mellitus (DM), coronary heart disease (CHD), chronic obstructive pulmonary disease (COPD), and hypertension have become the most common chronic diseases. China is the home to largest number of DM patients worldwide. It is estimated that there are currently 109.6 million adults living with DM, while in 2015 there were 1.3 million deaths caused by DM [2]. Furthermore, the economic burden of DM is substantial. In China, healthcare expenditures related to diabetes were 51 billion dollars in 2015, and they are expected to increase to 72 billion dollars by 2040 [2]. According to the fifth national health services survey in China, currently there are approximately 10.2 thousand CHD patients per million people [3], which is an increase of 34.5% in number of patients from 2008 [4]. COPD is characterized by chronic airflow limitation. It is a progressive lung disease and a leading cause of global death [5]. In China, the prevalence of COPD varies from 5 to 13% [6]. More than one billion people worldwide are diagnosed with hypertension, which is a NCD that causes stroke, heart disease, and kidney failure [7]. The hypertension prevalence rate is 14.3% among the population aged over 35 years or older in China [3]. The burden of disease among the aging population has become more serious than ever. In China, there are more than 0.23 billion people aged over 60, which accounted for 16.7% of the total population in 2016 [8]. The number of elderly people has increased by 29.5% since 2010 [9].

Following the shift from biomedicine model to the bio-psycho-social medical model [10], people have gained a deeper health awareness. Nowadays, the health measurements evaluate the life expectancy, as well as the quality of life (QoL). WHO has defined QoL as “the individual’s perception of their position in life in the context of the culture and value systems in which they live and in relation to their goals” [11]. The concept of health-related quality of life (HRQoL) can be interpreted as an indicator of individual’s well-being, and as effective pointer of potential health gains that can be brought on by various interventions [12]. The plan for “Healthy China 2030” was approved by the Political Bureau of the Communist Party of China Central Committee in 2016. Health promotion is an important part of national development strategy, and will remain so for at least next 15 years. Meanwhile, the healthcare reform in China is ever more comprehensive, thus improving the HRQoL in the whole population is one of its most important goals.

Patients’ health-related preferences have an important role for exploring their disease progression and survival, while health utility can be used to represent individual’s preference for a particular health state, which is widely used in health-related research and cost-utility analysis [13]. There are several health utility generic instruments, which mainly include the EuroQol 5-Dimensions (EQ-5D) [14], Health Utilities Index (HUI) [15], and Short Form-6 Dimensions (SF-6D) [16] questionnaires. The three-level version EQ-5D questionnaire (EQ-5D-3L) was introduced by EuroQol Group in 1990. EQ-5D-3L has been recommended by both the UK National Institute of Health and Care Excellence (NICE) and China Guidelines for Pharmacoeconomic Evaluations (2011 edition) as a preferred outcome measure tool [17, 18].

EQ-5D-3L comprises five dimensions, including “Mobility,” “Self-Care,” “Usual Activities,” “Pain/Discomfort,” and “Anxiety/Depression.” The questionnaire is divided in dimensions, and each dimension has three levels: “have no problems/be not,” “have some/moderate problems,” “have extremely problems/unable to.” Therefore, 3L questionnaire can be used to define 243 kinds of different health states [19]. Based on a value set, we can convert EQ-5D states to a single summary index, namely health utility, which can be used to calculate the Quality-adjusted life years (QALYs). The estimation of EQ-5D-3L value set is based on local people’s health preference and is affected by culture, social environment, as well as economic development. Thus, it is necessary to derive country-specific value set for EQ-5D health states. Since 1997, EQ-5D-3L value sets have been estimated by more than 20 countries (China, UK, USA, Korea, Japan, etc.). The questionnaire is currently translated into more than 170 languages, and is widely applied with good reliability and validity in both disease population (diabetes mellitus, hypertension, coronary heart disease, chronic obstructive pneumonia disease, etc.) and general population [20–23].

Due to the rising burden of diseases, it is necessary to pay more attention to HRQoL [24]. HRQoL can reveal the comprehensive survival state of a patient, and thus can provide more evidence for decision-makers, especially for chronic non-communicable diseases (CNCDs). In recent years, 3L questionnaire has been widely used in Chinese population with CNCDs to measure HRQoL. However, there is a lack of systematic reviews of these studies. The objective of the present review was to identify the kind of CNCD in China that EQ-5D-3L is mostly used for, as well as the variation of health utilities in different studies involving a specific CNCD.

## Methods

### Search strategy and selection criteria

We performed a systematic review according to the Preferred Reporting Items for Systematic Reviews and Meta-Analyses (PRISMA) guidelines [25]. All relevant studies that reported HRQoL evaluated by EQ-5D-3L questionnaire in Chinese patients with CNCDs were searched in PubMed, Embase, Web of Science, Cochrane Library, VIP, WanFang Data, and CNKI databases up to April 12, 2018. Search terms included “quality of life,” “QoL,” “HRQoL,” “EQ-5D,” “EuroQol,” “five dimension,” “China,” “Chinese,” “Randomized Controlled Trial,” “RCT,” “diseases,” and “chronic non-communicable,” and they were combined using Boolean logic (details in Appendix Table 3). Expert consultation and hand-searching of reference lists from retrieved studies were employed to identify additional references. VIP, WanFang Data, and CNKI are the most commonly used Chinese databases, which covers more than 95% of Chinese literatures, including journal articles, doctoral dissertations, masters’ theses, conference papers, reference works, newspapers, patents, and laws.

Following the inclusion criteria, all the studies were cross-sectional researches in Chinese population with CNCDs that were conducted in China, that reported EQ-5D-3L scores about a specific CNCDs with or without comorbidity by applying a value set, and that were full-text available. In this review, CNCDs are defined as “Diseases or conditions that occur in, or are known to affect, individuals over an extensive period of time and for which there are no known causative agents that are transmitted from one affected individual to another.” [26], such as cancer, DM, and COPD. We also included studies where health utility was generated from different country’s value set in the same sample. Languages were restricted to English and Chinese. We excluded any study if it was a review, or an abstract that used general population, communicable disease population, non-Chinese population, or Chinese subjects who did not live in China; that was longitudinal survey, intervention effect evaluation; where the only multiple diseases synthetic utility value was reported or there was no utility; and that was unrelated to HRQoL.

### Data collection and quality assessment

Preliminary literature screening was performed by two authors independently based on the titles and abstracts. After title/abstract review, full-text articles were reviewed by two investigators to evaluate eligibility of studies for inclusion and to check the bibliography. Two researchers independently conducted data extraction from all included articles using a pre-formulated sheet. Publication details, data sources, sample size (gender), type of disease, mean age, comorbidities, EQ-5D health utilities, EQ-VAS scores, five-dimension results, full health ratio, and value set information were extracted. Disagreement was solved by a further discussion between reviewers. To extract more information, all the results were pooled into a customized sheet when different articles reported HRQoL from the same dataset.

We appraised methodological quality of each study using a 11-item cross-sectional study assessment checklist introduced by Agency for Health Research and Quality (AHRQ) [27]. Each item was assigned one response option from three alternative choices, “Yes/No/Unclear,” based on study description. “Yes” for any item equaled one point, while “No” or “Unclear” equaled zero points. AHRQ defined the quality level of each article, and was obtained by adding all the item scores. A total of 0–3 points meant low quality, 4–7 points moderate quality, and over 7 points signified high quality.

### Statistical analysis

The variations of mean utility values described in all studies were reported. Besides that, descriptive analysis of EQ-VAS score ranges and response for each EQ-5D dimension were undertook. We conducted all calculations using Microsoft Excel 2013.

## Results

**Fig. 1:**
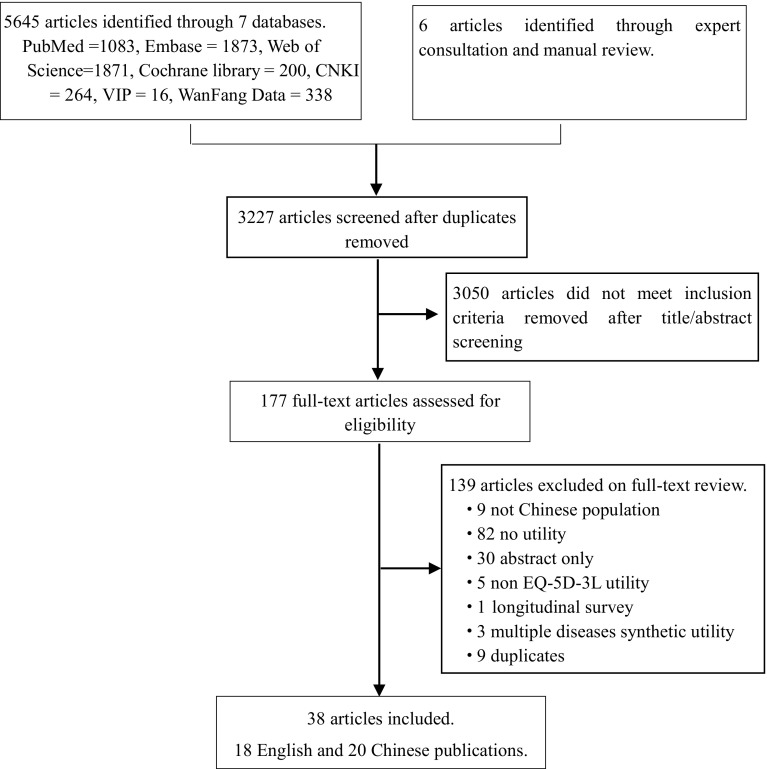
Flow diagram of article selection for inclusion

A total of 5027 English-language articles and 618 Chinese-language articles were identified via seven databases, while six additional studies were included after expert consultation and manual review. After checking for duplicates, we screened 3227 papers to assess for eligibility. Among these, 38 articles met the inclusion criteria [28–65] (Fig. 1). In total, 18 English-language articles and 20 Chinese-language articles were included in the review analysis. All the included cross-sectional studies were conducted between October 2006 and December 2017 (Table 1). Besides three studies that included only male patients [60, 63, 65], all the other studies included both male and female patients. The AHRQ checklist score ranged from 4 to 10 points, median score was 7 points, while mode was 7 (details in Appendix Table 4). There was no study of a low quality, while 29 studies were of median quality [29–31, 33–37, 39–42, 45–52, 54, 55, 57–59, 61, 62, 64, 65] and 9 were of high quality [28, 32, 38, 43, 44, 53, 56, 60, 63].

**Table 1:** Basic characteristics of included studies

	Survey time	Location	Patients	Male (%)	Disease	Mean age (SD), years	AHRQ score
Zhu [28]	2010	23 provinces	9650	51.0	T2DM	60.1 (11.7)	8
Liang [29]	December 2010 to January 2012	Beijing city	516	45.9	T2DM	62.3	5
Luo et al. [30]	July to October 2008	Nanjing city	256	50.4	T2DM	63.2 (9.9)	6
Tang et al. [31]	March 2014 to August 2014	Deqing county	415	55.9	T2DM	57.2 (16.6)	5
Han et al. [32]	December 2008 to July 2009	9 cities	7082	51.1	T2DM	59.6	8
Chang [33]	October 2006 to June 2007	Taiwan	498	45.8	T2DM	63.7 (13.8)	7
Yan et al. [34]	November 2007 to July 2012	Hong Kong	10,952	56.1	T2DM Normal ABI	58.2 (11.3)	7
			1230	45.1	T2DM Borderline ABI	60.4 (14.2)	
			590	47.1	T2DM PAD	68.3 (13.3)	
Ji et al. [35]	October 2011 to March 2012	China	998	49.6	T2DM Normal BMI	56.6	6
			822	49.3	T2DM Overweight BMI	56.5	
			212	33.0	T2DM Obese BMI	53.5	
Zhu et al. [36]	–	Ningbo city	319	–	Diabetes mellitus	50.7 (17.31)^a^	4
			1383	–	Hypertension		
			45	–	COPD		
			41	–	Stroke		
Cao et al. [37]	August to October 2010	Beijing city	802	27.9	Diabetes mellitus	57.2 (9.77)^a^	5
			3263	34.7	Hypertension		
			416	44.0	Stroke		
			1930	28.0	Coronary heart disease		
Xiong et al. [38]	August 2007 to January 2010	Nanchang city	330	65.2	Coronary heart disease	65.4 (10.8)	8
Wang et al. [39]	August to October 2010	Beijing city	1928	29.4	Coronary heart disease	61.6 (9.2)	7
Wu et al. [40, 41]^b^	July to December 2011	Tianjing and Chengdu city	411	49.6	Chronic stable angina	68.1 (11.4)	7
Wu et al. [42]	March to June 2011	Beijing, Guangzhou, Shanghai and Chengdu city	678	72.9	COPD	70.4 (10.1)	7
Chen et al. [43]	September 2010 to May 2011	Hong Kong	154	98.7	COPD	72.9 (8.1)	8
Ding et al. [44]	2009	China	675	60.7	COPD	62.0 (11.4)	8
Gao et al. [45]	July to October 2012	Wuhan city	144	52.1	Epilepsy	33.1 (13.0)	6
Gao et al. [46]	July 2012 to January 2013	Wuhan city	220	53.6	Epilepsy	31.8 (13.0)	5
Li et al. [47]	2011 to 2012	Hangzhou and Beijing city	1006	–	Hypertension	–	6
He et al. [48]	December 2011 to February 2012	Beijing city	606	38.8	Hypertension	65.9	4
Wang 2017 [49]	July to September 2017	Lian-yungang city	2125	43.2	Hypertension	59.5 (9.2)	7
Wang et al. [50]	January to December 2017	Dalian city	487	48.5	Hypertension	65.6 (6.7)	5
Zhang et al. [51]	2014	Shanghai city	419	46.3	Hypertension	–	7
He et al. [52]	–	Baoji city	123	58.5	Cerebral infarction	58.6 (13.2)	4
Wei [53]	November 2012 to March 2013	Guangxi Autonomous Region	60	60.0	Cerebral infarction DBP	57.5 (10.1)	10
			94	66.0	Cerebral infarction NDBP	61.6 (9.8)	
			99	67.7	Cerebral infarction ADBP	66.3 (9.4)	
Che et al. [54]	December 2012 to June 2013	Kunming city	91	84.6	Compensated	48 (11.3)	6
			198	77.8	Decompensated	49 (11.8)	
			131	79.4	HCC	56 (11.1)	
			100	75.0	Liver failure	44 (12.3)	
Yu et al. [55]	August to October 2015	Beijing city	55	81.8	Compensated	50.9 (1.6)	6
			64	68.8	Decompensated	52.4 (1.4)	
			45	77.8	HCC	58.4 (1.7)	
Chen [56]	December 2014 to July 2015	Anhui province	188	68.6	Lung cancer	26–85^c^	8
Chen et al. [57]	December 2014 to July 2015	Anhui province	209	78.0	Esophagus cancer	43–89^c^	7
Cui [58]	2008	Heibei province	340	63.8	cerebral palsy	7.8 (2.3)	6
Gu [59]	July 2008 to January 2009	Shanghai city	92	10.9	Rheumatoid arthritis	52.5 (12.3)	7
Jiang [60]	September 2015 to January 2016	Shandong province	42	100.0	Sarcopenia	68.7 (8.0)	9
Wang et al. [61]	October 2009 to May 2010	Taiwan	742	59.8	Atrial fibrillation	70.2 (11.8)	7
Farooq et al. [62]	June to December 2009	Shaanxi province	368	48.6	Kashin beck disease	56.9 (10.1)	6
Zhao et al. [63]	December 2008 to March 2009	Kunming city	268	100.0	Chronic prostatitis	33.2 (8.0)	9
Lin et al. [64]	January to May 2008	Taipei city	318	48.1	Visual impairment	74	7
Sun et al. [65]	June 2011 to February 2012	China	110	100.0	Hemophilia	30.4 (7.8)	6

*SD* standard deviation, *AHRQ* agency for health research and quality, *T2DM* type 2 diabetes mellitus, *ABI* ankle-brachial index, *PAD* peripheral arterial disease, *BMI* body mass index, *COPD* chronic obstructive pulmonary disease, *DBP* dipper blood pressure, *NDBP* non-dipper blood pressure, *ADBP* anti-dipper blood pressure, *HCC* hepatocellular cancer, – not reported in excluded study

^a^Full sample’ mean age and SD

^b^Same sample applied two different value sets in two articles, respectively

^c^Only reported age range

We extracted HRQoL data on 18 kinds of CNCDs based on EQ-5D-3L from the included studies (Table 2). The diseases were diabetes mellitus (DM), hypertension, coronary heart disease (CHD), chronic obstructive pneumonia disease (COPD), epilepsy, cerebral infarction (CI), stroke, chronic liver diseases (CLD), lung cancer (LC), esophagus cancer (EC), cerebral palsy (CP), rheumatoid arthritis (RA), sarcopenia, atrial fibrillation (AF), Kashin Beck disease (KBD), chronic prostatitis (CPT), visual impairment (VD), and hemophilia.

**Table 2:** HRQoL of Chinese disease population based on EQ-5D-3L

	Disease	Health utility		VAS scores		Have some/extremely problems in 5 dimensions (%)					Full health (%)	Value set	Administration
		Mean value	SD	Mean value	SD	Mobility (%)	Self-care (%)	Usual activities (%)	Pain/discomfort (%)	Anxiety/depression (%)			
Diabetes mellitus													
Zhu [28]	T2DM	0.81	0.08	78.6	11.4	6.9	4.0	6.1	19.5	15.6	71.0	Japan	Face-to-face
Liang [29]	T2DM	0.85	–	73.9	–	13.0	2.5	7.0	42.4	25.6	–	Japan	Face-to-face
Luo et al. [30]	T2DM	0.79	0.16	–	–	21.5	7.8	41.8	41.8	36.7	–	Japan	Face-to-face
Tang et al. [31]	T2DM	0.84	0.20	61.5	16.5	21.2	11.5	17.3	38.5	47.1	36.5	China	Face-to-face
Han et al. 2013 [32]	T2DM	0.87	0.21	71.0	14.6	15.5	8.8	14.4	26.8	26.8	56.7	UK	Face-to-face
Chang [33]	T2DM	0.80	0.20	–	–	–	–	–	–	–	–	UK	Face-to-face
Yan et al. [34]	T2DM normal ABI^a^	0.90	–	–	–	1.7	3.7	5.9	20.9	26.8	–	UK	NA
	T2DM borderline ABI^b^	0.88	–	–	–	3.8	7.8	12.0	22.6	29.1	–	UK	NA
	T2DM PAD^c^	0.80	–	–	–	14.0	21.9	33.6	23.2	36.4	–	UK	NA
Ji et al. [35]	T2DM normal BMI^d^	0.90	–	–	–	9.6	6.3	15.6	26.7	16.6	–	UK	Self-administered
	T2DM overweight BMI^e^	0.85	–	–	–	14.2	8.1	21.4	39.7	23.8	–	UK	Self-administered
	T2DM obese BMI^f^	0.81	–	–	–	14.3	7.1	23.4	58.4	29.9	–	UK	Self-administered
Zhu et al. [36]	Diabetes mellitus	0.80	0.15	–	–	–	–	–	–	–	–	Japan	Face-to-face
Cao et al. [37]	Diabetes mellitus	0.94	0.14	–	–	15.0	7.9	13.5	22.2	8.1		Japan	Face-to-face
Hypertension													
Li et al. [47]	Hypertension	0.80	0.17	–	–	–	–	–	–	–	–	UK	Face-to-face
He et al. [48]	Hypertension	0.78	0.19	77.4	14.4	–	–	–	–	–	–	UK	NA
Wang [49]	Hypertension	0.84	0.22	70.1	19.0	15.4	4.8	9.9	45.2	16.8	–	UK	Face-to-face
Wang et al. [50]	Hypertension	0.91	0.15	71.0	14.6	8.0	3.1	6.6	26.1	13.1	–	Japan	Face-to-face
Zhu et al. [36]	Hypertension	0.80	0.13	–	–	–	–	–	–	–	–	Japan	Face-to-face
Cao et al. [37]	Hypertension	0.93	0.14	–	–	14.6	8.4	13.0	20.2	7.4	–	Japan	Face-to-face
Zhang et al. [51]	Hypertension	0.92	0.17	–	–	6.8	3.6	6.5	11.3	5.8	–	China	Face-to-face
Coronary heart disease													
Cao et al. [37]	Coronary heart disease	0.90	0.16	–	–	17.7	9.4	15.4	24.2	8.1		Japan	Face-to-face
Xiong et al. [38]	Coronary heart disease	0.86	0.15	77.5	13.8	–	–	–	–	–	–	Japan	Telephone
Wang et al. [39]	Coronary heart disease	0.89	0.17	71.6	17.7	17.9	9.5	15.5	24.3	7.9	–	Japan	Face-to-face
Wu et al. [40]	Chronic stable angina	0.78	0.15	71.2	12.4	15.8	13.4	–	–	–	15.6	China	Face-to-face
Wu et al. [41]	Chronic stable angina	0.75	–	71.2	–	15.8	13.4	60.3	55.7	56.0	15.6	UK	Face-to-face
COPD													
Zhu et al. [36]	COPD	0.76	0.15	–	–	–	–	–	–	–	–	Japan	Face-to-face
Wu et al. [42]	COPD	0.73	0.15	66.6	16.2	39.1	17.3	37.8	38.0	29.4	–	Japan	Face-to-face
Chen et al. [43]	COPD	0.64	0.31	55.3	20.4	–	–	–	–	–	22.1	UK	Face-to-face
Ding et al. [44]	COPD	0.80	0.30	–	–	–	–	–	–	–	–	–	Face-to-face
Epilepsy													
Gao et al. [45]	Epilepsy	0.83	0.21	79.6	16.4	7.6	7.6	15.3	34.7	47.9	–	UK	Face-to-face
Gao et al. [46]	Epilepsy	0.87	0.24	78.3	15.8	–	–	–	–	–	–	UK	Face-to-face
Cerebral infarction													
He et al. [52]	Cerebral infarction	0.53^j^	–	66.8	14.8	22.0	14.6	23.6	47.2	25.2	–	UK	Face-to-face
Wei [53]	Cerebral infarction DBP^g^	0.75	0.08	79.0	23.5	–	–	–	–	–	–	Japan	Face-to-face
	Cerebral infarction NDBP^h^	0.62	0.12	64.9	18.4	–	–	–	–	–	–	Japan	Face-to-face
	Cerebral infarction ADBP^i^	0.51	0.11	49.7	17.0	–	–	–	–	–	–	Japan	Face-to-face
Stroke													
Zhu et al. [36]	Stroke	0.51	0.33	–	–	–	–	–	–	–	–	Japan	Face-to-face
Cao et al. [37]	Stroke	0.90	0.17	–	–	21.2	13.5	20.7	24.0	10.1	–	Japan	Face-to-face
Chronic liver disease													
Che et al. [54]	Compensated	0.70	0.20	58.2	14.9	–	–	–	–	–	–	Thailand	Face-to-face
Yu et al. [55]	Compensated	0.80	0.03	–	–	–	–	–	–	–	–	Japan	Self-administered
Che et al. [54]	Decompensated	0.60	0.30	47.6	23.4	–	–	–	–	–	–	Thailand	Face-to-face
Yu et al. [55]	Decompensated	0.63	0.05	–	–	–	–	–	–	–	–	Japan	Self-administered
Che et al. [54]	Liver failure	0.00	0.20	36.4	17.2	–	–	–	–	–	–	Thailand	Face-to-face
Che et al. [54]	HCC	0.60	0.30	50.6	16.9	–	–	–	–	–	–	Thailand	Face-to-face
Yu et al. [55]	HCC	0.41	0.07	–	–	–	–	–	–	–	–	Japan	Self-administered
Other diseases													
Chen [56]	Lung cancer	0.79	0.25	73.6	13.9	24.5	12.8	26.6	47.3	30.9	–	UK	Face-to-face
Chen et al. [57]	Esophagus cancer	0.84	0.22	75.2	11.0	18.2	12.0	22.0	38.3	25.4	48.8	UK	NA
Cui [58]	Cerebral palsy	0.44	0.12	27.3	9.1	87.8	94.3	94.3	58.4	72.1	–	Japan	Face-to-face
Gu [59]	Rheumatoid arthritis	0.56	0.30	–	–	–	–	–	–	–	–	UK	Face-to-face
Jiang [60]	Sarcopenia	0.78	–	78.8	–	21.4	7.1	9.5	50.0	19.1	–	UK	Face-to-face
Wang et al. [61]	Atrial fibrillation	0.81	0.25	70.3	14.4	27.5	37.3	22.9	12.5	21.6	–	–	Face-to-face
Farooq et al. [62]	Kashin–beck disease	0.45	0.30	60.5	18.0	76.1	57.1	69.3	89.9	75.8	–	UK	Face-to-face
Zhao et al. [63]	Chronic prostatitis	0.73	0.15	69.2	14.2	3.0	0.0	6.3	82.1	69.4	–	UK	Face-to-face
Sun et al. [64]	Hemophilia	0.71	0.20	71.0^k^	21.0^k^	71.8	27.3	58.2	65.5	60.0	–	USA	Web-based
Lin et al. [65]	Visual impairment	0.85	–	–	–	23.6	10.4	20.1	43.7	39.3	–	–	Face-to-face

*VAS* visual analogue scale, *SD* standard deviation, *T2DM* type 2 diabetes mellitus, *ABI* ankle-brachial index, *PAD* peripheral arterial disease, *BMI* body mass index, *COPD* chronic obstructive pulmonary disease, *DBP* dipper blood pressure, *NDBP* non-dipper blood pressure, *ADBP* anti-dipper blood pressure, *HCC* hepatocellular cancer, *NA* not available, *HRQoL* health-related quality of life, *EQ-5D-3L* 3 level version of EuroQol 5-Dimensions, – not reported in excluded study

^a^Normal ABI: 1.00 < ABI ≤ 1.40

^b^Borderline ABI: 0.9 < ABI ≤ 0.99

^c^PAD: ABI ≤ 0.9.

^d^Normal BMI: 18.5 ≤ BMI < 24.0

^e^Overweight BMI: 24.0 ≤ BMI < 28.0

^f^Obese BMI: 28.0 ≤ BMI

^g^DBP 10%≤Nocturnal Reduction Rate ≤ 20%

^h^NDBP: 10% > Nocturnal Reduction Rate

^i^ADBP: 20% < Nocturnal Reduction Rate.

^j^Only reported median utility value

^k^The original data were scaled in 10-point system

### Diabetes mellitus

In this review, ten studies reported health utilities for diabetes mellitus [28–37]. The extreme values as well as the utility values that ranged from 0.79 to 0.94 were calculated by Japanese value set. However, the study with the highest values [37] was conducted in rural communities and reported a younger mean age (57.2 vs. 63.2 years) without any comorbidity compared to the study that was conducted at a hospital and that described a few serious T2DM comorbidities (hyperlipidemia, cardiovascular disease, and hypertension) with the lowest value [30]. Interestingly, when applied in Chinese value set, the results from Tang’s study that included about 415 T2DM patients was 0.84 [31]. The EQ-5D VAS scores were from 61.5 to 78.6 in four studies [28, 29, 31, 32]. The decrease of health utility in DM patients was mainly caused by problems related to “Pain/Discomfort” and “Anxiety/Depression” dimensions. Hypertension, hyperlipemia, and CHD were the most common DM comorbidities reported by the studies, and the prevalence of DM comorbidities was from 42.6 to 81.5%, thus having a significant influence on HRQoL.

### Hypertension

For the patients with hypertension, the utility values ranged from 0.78 to 0.93 in six studies [36, 37, 47–51]. Japanese value set and UK value set were applied in the hypertension disease population in the studies that reported the highest value [37] and the lowest value [48], respectively. We found that the study [37] with the highest value reported a younger mean age without any comorbidity compared to the study [48] on patients with hypertension and comorbidities. The EQ-5D VAS scores were from 70.1 to 77.4 in three studies [48–50]. “Pain/Discomfort” was the dimension with the most problems reported by the patients in three studies [37, 49, 50].

### Coronary heart disease

For the patients with CHD, the utility values ranged from 0.75 to 0.90 in five studies [37–41]. Two of them were about chronic stable angina (CSA) patients, which was a subgroup of CHD [40, 41]. The extreme values were generated by UK [41] (0.75) and Japanese value set [37] (0.90), respectively. In general, the mean age of CHD patients with highest utility was 57.2 years old and 68.1 years for those with the lowest utility. Moreover, the former was concerned with CHD patients without comorbidity in rural areas [41], while the latter included more serious CSA patients with comorbidities at hospitals [36]. Chinese and UK values have been separately applied in the same CHD sample in a previous study by Wu et al. [30, 41], and health utility calculated by Chinese value set [40] (0.78) was a little bit higher compared to UK set [41] (0.75). In terms of EQ-5D VAS scores, they ranged from 71.2 to 77.5 in four studies [38–41]. “Pain/Discomfort” was the dimension with the most problems reported by CHD patients in two studies [36, 38], while “Usual Activities” in CSA patients [40, 41]. Prevalence of comorbid hypertension most commonly occurred among CHD patients, followed by DM [37].

### Chronic obstructive pneumonia disease

The health utility values for COPD patients ranged from 0.64 to 0.80 in four studies [36, 42–44]. The lowest value was calculated by UK value set [43]; however, the study that reported highest value did not describe the value set applied [44]. Patients with the highest value had a younger mean age and better post-bronchodilator FEV_1_ of predicted than the lowest one. Two studies reported that EQ-5D VAS scores were 55.3 [43] and 66.6 [42], respectively. The decrease of health utility in COPD patients was mainly caused by problems in “Mobility” dimension that were only described in one study [42]. The prevalence of comorbidities in COPD patients was from 67.5 to 78.9% [42, 43].

### Epilepsy

The health utility values for epilepsy patients ranged from 0.83 to 0.87 in two studies [45, 46], and both were calculated by UK value set. The patients in the study [46] that reported a higher utility were a little bit younger compared to patients in another study [45]. Besides that, it is possible that disease duration negatively affects the utility, since mean epilepsy duration of 8.5 years was reported in the study with lower value [45] compared to 6.0 years reported by another study [45]. EQ-5D VAS scores were 78.3 [46] and 79.6 [45], respectively. “Anxiety/Depression” was the most problematic dimension followed by “Pain/Discomfort” [45].

### Cerebral infarction

In terms of health utility for patients with CI, two studies reported the HRQoL [52, 53]. Among these, one included three subgroup analyses based on different types of blood pressure [53]. The utility values and VAS scores were much lower in anti-dipper blood pressure group (0.51/49.7) compared to dipper blood pressure group (0.75/79.0). The decrease of health utility in CI patients was mainly caused by problems in “Mobility” dimension [52].

### Stroke

For the patients with stroke, the health utility ranged from 0.51 to 0.90 in two studies evaluated by Japanese value set [36, 37]. The wide range of utility values for stroke was caused by the variation in mean age, comorbidities, and disease severity, etc. “Pain/Discomfort” was the dimension with most problems followed by “Mobility” [37]. No information was available on EQ-5D VAS score.

### Chronic liver disease

The health utility values for patients with CLD differed in disease severity. The values ranged from 0.70 to 0.80 for compensated patients [54, 55], while it ranged from 0.60 to 0.63 for decompensated patients [54, 55]. When the disease deteriorated to HCC, utility values were from 0.41 to 0.60 [54, 55], which was lower compared to compensated or decompensated patients. In addition, the health utilities of liver failure were 0.00 [54] which was equal to death. In terms of EQ-5D VAS scores, they ranged from 36.4 [54] (for liver failure) to 58.2 [54] (for compensated) in the study. There were no results on the most problematic dimension in CLD patients.

### Other diseases

For the remaining ten diseases [56–65], i.e., lung cancer, sarcopenia, and hemophilia, the health utility value for each disease was only reported by one study.

Among the ten diseases, cerebral palsy was 0.44 [58] for the utility value which was the lowest one, while the highest one was 0.85 [64] in visually impaired patients. Japanese value set was applied in cerebral palsy patients. In terms of EQ-5D VAS scores, they ranged from 27.3 [58] (cerebral palsy) to 78.8 [60] (sarcopenia). “Pain/Discomfort” or “Anxiety/Depression” was the dimensions that caused most problems according to majority of studies.

## Discussion

The present review focused on HRQoL in chronic non-communicable diseases in Chinese population. Over recent years, EQ-5D-3L questionnaire has been increasingly applied in different patient groups in China to measure their health utility values. Among 18 different types of diseases, DM, CHD, COPD, and hypertension are the most common CNCDs in China. Due to the high morbidity and mortality rates from these CNCDs, people have become more than ever concerned about the patients’ state of survival and HRQoL.

Patient-reported outcomes are important to health decision-makers. As a generic instrument, EQ-5D can be easily used by patients to report their HRQoL. However, there are variations in health utility values for a specific CNCD among different studies. Given the level of heterogeneity is high regarding patient characteristics and study design, meta-analysis is not an appropriate method to calculate a single index across studies. The utility values of DM (0.79–0.94), CHD (0.75–0.90), COPD (0.64–0.80), hypertension (0.78–0.93), epilepsy (0.83–0.87), CI (0.51–0.75), stroke (0.51–0.90), and CLD (0.00–0.80) reflect HRQoL in patients with CNCDs and with different conditions in a QALY framework. The results can be changed by a series of factors, including age, gender, sample source, comorbidities, rural/urban, and value set. In general, the health status deteriorates as people get old. Thus, the utility value decreases with the increasing age. According to previous study, the values in patients with T2DM aged 60 and over (0.83) was lower compared to patients with T2DM who were younger than 60 (0.86) [23].

In most of the studies that reported on gender-specific health utility values and were included in the present review [28–30, 38, 39, 48, 49, 56, 57] men had a better HRQoL compared to women, e.g., with reference to lung cancer, man had 0.81, whereas woman had 0.76 value [56]. These results are in line with what has observed in the general population that men have a higher mean EQ-5D value score than women [23, 66]. Besides gender, community-based or hospital-based cross-sectional surveys also have influence on the HRQoL assessment. It is logical to expect that patients in hospital will report more problems compared to stay at home patients. In line with previous statement, Chen et al. [43] conducted a survey to measure health utility in patients with COPD at hospital, while Zhu et al. [36] conducted the same survey in community, and the values have shown to lower in the former sample.

Comorbidity has an important role in the variation of health utility value. In addition to the number of comorbidities, different types of comorbidities can affect health utility values as well. Hypertension, DM, CHD, hyperlipidemia, and stroke are the most common comorbid conditions [28, 30, 36–38] The HRQoL in patients who do not have comorbidities with other diseases is better compared to the patients with comorbidities. Luo has reported that the value of utility in people only suffering DM was 0.86; however, it dropped to 0.69 when there were other comorbid conditions present [30]. Thus, the value of utility decreases in the presence of other comorbid diseases. In Liang’s study [29], DM patients with one, two, or more than two kinds of comorbidities revealed the utility value of 0.86, 0.83, and 0.81, respectively. Moreover, various comorbidities have different interaction effects on health utility. When patients have different kinds of comorbid conditions, HRQoL may change. According to Wang, patients with CHD and hypertension, and DM or stroke have the utility values of 0.89, 0.87, and 0.85, respectively. Stroke is a serious comorbidity in many diseases [32, 37, 39, 67].

China is a country with dual economic structure between rural and urban areas [68] Due to the special economic structure, social policy and welfare are different for citizens living in city and countryside, and thus the medical service system and social insurance may have an impact on HRQoL. With reference to the impact of urban/rural context on HRQoL, it is still a matter of some controversy. Chen has reported that EC patients living in rural areas have a higher health utility value compared to those living in urban areas [57]. However, as regards to people with LC, there has been no difference between rural and urban areas [56]. In China, most rural people are covered by “New Rural Cooperative Medical Insurance (NRCMI),” while urban people are covered by “Urban Residents Basic Medical Insurance (URBMI)” and “Urban Employee Basic Medical Insurance (UEBMI).” By the end of 2015, there were approximately 670 million, 377 million, 289 million people enrolled in NRCMI, URBMI, and UEBMI, respectively [69]. However, medical resources are distributed unequally, most of which are allocated in tertiary hospitals in urban areas. Furthermore, larger gap exists in terms of quality of medical services between urban and rural areas. Su et al. [70] compared the effects of NRCMI, URBMI, and UEBMI on HRQoL, and the results showed that the insured people of UEBMI had a higher mean EQ-5D utility score. Besides that, the horizontal inequality index suggested that there existed a higher pro-rich health inequity in NRCMI than urban schemes.

The application of value sets from various countries in the same disease population leads to different results in health utility values. In the same sample of patients with CHD [40], the values reported by Chinese value set were higher compared to UK value set [41]. The estimations of EQ-5D-3L value sets are based on local people’s health preference and are affected by culture, social environment, and economic development. Furthermore, the preference in health might vary across different countries. Time trade-off is the most widely accepted method for estimating a EQ-5D-3L value set. Respondents are asked to imagine a certain health condition described by EQ-5D-3L that would be experienced for a fixed time (e.g., 10 years) and then to compare it with a shorter time in full health. Five countries’ value sets (Japan [71], China [72], UK [73], Thailand [74], USA [75]) applied in the included studies are showed in Appendix Table 5. The best ill health state value is 0.961 with Chinese value set, higher than other four countries’, which indicates the departure from full health declines less in health state value. “Pain/Discomfort” and “Anxiety/Depression” dimensions have a larger impact on disutility when applied in UK and USA value sets, while “Usual Activities” in Chinese value set. Chinese EQ-5D-3L value set has been estimated in 2014 [72], and it has shown to be the most appropriate set to use for exploration of the HRQoL in disease or general population in China.

EQ-5D-3L may lead to ceiling effects when measure HRQoL and health decrements may not be sensitive in disease population [76]. Five-level version (EQ-5D-5L) was introduced by EuroQol Group in 2005 [77] to reduce ceiling effects and improve the questionnaire’s sensitivity to mild changes in health that cannot be capture by EQ-5D-3L. Both the EQ-5D-3L and EQ-5D-5L comprise the same five dimensions, but EQ-5D-5L is added two more levels in each dimension: “have no problems/be not,” “have slight problems/be slightly,” “have some/moderate problems,” “have severe problems/be severely,” “have extremely problems/unable to.” Therefore, EQ-5D-5L can define 3125 kinds of different health states. Although new version of EQ-5D questionnaire has some advantages over old version, EQ-5D-5L value sets have only been published since 2016. Chinese EQ-5D-5L value set has recently been estimated in 2017 [78]. The broad application of EQ-5D-5L country-specific value sets are limited by the publication time. Most of researchers are unfamiliar with the new value sets. In view of this, when conducting a health economic assessment or population survey, researchers are still accustomed to using EQ-5D-3L to measure health utilities.

Compared with other countries’ patients, the health utility of European people with T2DM was 0.69 and it was 0.65 in New Zealand and Australia [79, 80]. In general, the HRQoL of Chinese T2DM patients might be better than these countries’ T2DM sufferers. A systematic review reported that the utility values of cardiovascular disease patients ranged from 0.24 to 0.90 [81], and the highest EQ-5D-3L values were reported in people living with CHD. For COPD patients, a meta-analysis reported the utility values were from 0.62 to 0.82 by severity of the disease [82], and the results were similar to COPD patients in China.

The main limitation of this review is number of studies reporting on each CNCD. Even though 18 different kinds of diseases were included, more than half of the CNCDs were reported separately. Due to the lack of sufficient information on health utility for some of the CNCDs discussed above, it is difficult to get accurate conclusions about the HRQoL in various Chinese population with CNCDs.

The comparison and analysis of HRQoL across different populations with CNCDs is of utmost importance. Utility value is a single index that reflects synthetic information about people’s health, and that can provide useful evidence for decision-makers upon optimizing the allocation of health resources.

## Appendix

See Tables 3, 4, 5.

**Table 3:** Literature search strategies

	Search terms	Databases			
		PubMed	Embase	Web of science	Cochrane library
#1	Quality of life	218,659	385,677	388,562	75,309
#2	QoL	30,551	56,702	28,499	10,349
#3	HRQoL	12,774	20,084	12,350	3340
#4	#1 or #2 or #3	220,618	392,362	390,234	76,062
#5	eq-5d	5825	10,988	6259	3390
#6	EuroQol	4051	6057	4298	2277
#7	Five dimension	154	5058	26,161	628
#8	#5 or #6 or #7	7754	18,375	33,795	4533
#9	#4 and #8	5691	11,107	7616	3698
#10	China	128,958	153,634	488,511	40,413
#11	Chinese	170,044	221,032	326,135	49,739
#12	#10 or #11	267,060	335,522	733,456	71,681
#13	#9 and #12	140	273	220	284
#14	#4 and #12	4284	7302	8980	5892
#15	#8 and #12	195	444	1023	302
#16	#14 or #15	4339	7473	9783	5903
#17	RCT	16,672	27,771	16,305	512,063
#18	Randomized controlled trial	53,410	295,111	387,155	628,242
#19	Clinical trial	118,324	326,333	582,836	688,438
#20	#17 or #18 or #19	177,958	502,861	806,195	983,165
#21	#14 not #20	3887	6313	7325	491
#22	#15 not #20	183	418	980	9
#23	#13 not #20	136	257	194	10
#24	#21 or # 22	3937	6474	8111	491
#25	Disease	2,696,747	3,686,979	3,592,538	266,098
#26	Chronic non-communicable	566	729	1390	136
#27	#25 or #26	2,697,028	3,687,355	3,592,546	266,115
#28	#16 and #27	1189	2177	2489	3537
#29	#24 and #27	1083	1873	1871	200

**Table 4:** AHRQ checklist scoring

	1	2	3	4	5	6	7	8	9	10	11	Scores
Zhu [28]	Y	Y	Y	UC	N	Y	Y	Y	Y	Y	UC	8
Liang [29]	Y	Y	Y	UC	N	Y	N	Y	N	N	UC	5
Luo et al. [30]	Y	Y	Y	Y	N	Y	N	Y	N	N	UC	6
Tang et al. [31]	Y	Y	Y	UC	N	Y	N	Y	N	N	UC	5
Han et al. [32]	Y	Y	Y	Y	Y	Y	N	Y	N	Y	UC	8
Chang [33]	Y	Y	Y	N	Y	N	Y	Y	N	Y	UC	7
Yan et al. [34]	Y	Y	Y	UC	Y	N	Y	Y	N	Y	UC	7
Ji et al. [35]	Y	Y	Y	UC	N	Y	Y	N	Y	N	UC	6
Zhu et al. [36]	Y	Y	N	UC	N	Y	N	Y	N	N	UC	4
Cao et al. [37]	Y	Y	Y	UC	N	Y	N	Y	N	N	UC	5
Xiong et al. [38]	Y	Y	Y	Y	N	Y	N	Y	Y	N	Y	8
Wang et al. [39]	Y	Y	Y	UC	Y	Y	N	Y	N	Y	UC	7
Wu et al. [40]	Y	Y	Y	UC	Y	Y	N	Y	N	Y	UC	7
Wu et al. [41]	Y	Y	Y	UC	N	Y	Y	Y	N	Y	UC	7
Wu et al. [42]	Y	Y	Y	Y	N	Y	N	Y	N	Y	UC	7
Chen et al. [43]	Y	Y	Y	Y	N	Y	Y	Y	N	Y	UC	8
Ding et al. [44]	Y	Y	Y	Y	Y	Y	N	Y	N	Y	UC	8
Gao et al. [45]	Y	Y	Y	UC	Y	Y	N	Y	N	N	UC	6
Gao et al. [46]	Y	Y	Y	UC	N	Y	N	Y	N	N	UC	5
Li et al. [47]	Y	Y	Y	UC	N	Y	N	Y	N	Y	UC	6
He et al. [48]	Y	N	Y	UC	N	Y	N	N	N	Y	UC	4
Wang [49]	Y	Y	Y	Y	Y	Y	N	Y	N	N	UC	7
Wang et al. [50]	Y	Y	Y	UC	N	Y	N	Y	N	N	UC	5
Zhang et al. [51]	Y	Y	Y	Y	N	Y	N	Y	N	Y	UC	7
He et al. [52]	Y	Y	N	UC	Y	Y	N	N	N	N	UC	4
Wei [53]	Y	Y	Y	Y	Y	Y	Y	Y	N	Y	Y	10
Che et al. [54]	Y	Y	Y	Y	N	Y	N	Y	N	N	UC	6
Yu et al. [55]	Y	Y	Y	Y	N	Y	N	Y	N	N	UC	6
Chen [56]	Y	Y	Y	Y	Y	Y	N	Y	N	Y	UC	8
Chen et al. [57]	Y	Y	Y	Y	N	Y	N	Y	N	Y	UC	7
Cui [58]	Y	Y	Y	UC	N	Y	N	Y	N	Y	UC	6
Gu [59]	Y	Y	Y	Y	Y	Y	N	Y	N	N	UC	7
Jiang [60]	Y	Y	Y	Y	Y	Y	Y	Y	N	Y	UC	9
Wang et al. [61]	Y	Y	Y	Y	Y	N	Y	N	N	N	UC	7
Farooq et al. [62]	Y	Y	Y	UC	N	Y	N	N	N	Y	UC	6
Zhao et al. [63]	Y	Y	Y	Y	Y	Y	Y	Y	N	Y	UC	9
Lin et al. [64]	Y	Y	Y	Y	N	Y	N	Y	N	N	Y	7
Sun et al. [65]	Y	Y	Y	UC	Y	Y	N	Y	N	N	UC	6

*Y* yes, *UC* unclear, *N* no, *AHRQ* agency for health research and quality. *AHRQ checklist items* 1—Define the source of information, 2—List inclusion and exclusion criteria for exposed and unexposed subjects or refer to previous publications. 3—Indicate time period used for identifying patients. 4—Indicate whether or not subjects were consecutive if not population-based. 5—Indicate if evaluators of subjective components of study were masked to other aspects of the status of the participants. 6—Describe any assessments undertaken for quality assurance purposes. 7—Explain any patient exclusions from analysis. 8—Describe how confounding was assessed and/or controlled. 9—If applicable, explain how missing data were handled in the analysis. 10—Summarize patient response rates and completeness of data collection. 11—Clarify what follow-up, if any, was expected and the percentage of patients for which incomplete data or follow-up was obtained

**Table 5:** The EQ-5D-3L value sets of five countries

	Japan [71]	China [72]	UK [73]	Thailand [74]	USA [75]
Constant	0.152	0.039	0.081	0.202	–
MO2	0.075	0.099	0.069	0.121	0.146
MO3	0.418	0.246	0.314	0.432	0.558
SC2	0.054	0.105	0.104	0.121	0.175
SC3	0.102	0.208	0.214	0.242	0.471
UA2	0.044	0.074	0.036	0.059	0.140
UA3	0.133	0.193	0.094	0.118	0.374
PD2	0.080	0.092	0.123	0.072	0.173
PD3	0.194	0.236	0.386	0.209	0.537
AD2	0.063	0.086	0.071	0.032	0.156
AD3	0.112	0.205	0.236	0.110	0.450
N3	–	0.022	0.269	0.139	–
D1	–	–	–	–	− 0.140
(I2)^2	–	–	–	–	0.011
(I3)	–	–	–	–	− 0.122
(I3)^2	–	–	–	–	− 0.015
Sample size	621	1147	3395	1409	4048
States directly valued	17	97	42	86	42
Valuation method	TTO	TTO	TTO	TTO	TTO
Range	(− 0.111, 0.804)	(− 0.149, 0.961)	(− 0.594, 0.883)	(− 0.452, 0.766)	(− 0.102, 0.860)

*mo* mobility, *SC* self-care, *UA* usual activities, *PD* pain/discomfort, *AD* anxiety/depression, *N3* any dimension is at level 3, *D1* the number of dimension not at level 1, minus 1, *I2* the number of dimension is level 2, minus 1, *(I2)^2* the square of I2, *I3* the number of dimensions at level 3, minus 1, *(I3)^2* the square of I3, *TTO* time trade-off
